# Lower limb arterial calcification and its clinical relevance with peripheral arterial disease

**DOI:** 10.3389/fcvm.2023.1271100

**Published:** 2023-11-24

**Authors:** Yue Dong, Yuankang Liu, Panpan Cheng, Hongli Liao, Cuiping Jiang, Ying Li, Shuhua Liu, Xiangyang Xu

**Affiliations:** ^1^ Department of Radiology, Liyuan Hospital, Tongji Medical College, Huazhong University of Science and Technology, Wuhan, China; ^2^ Department of Radiology, The Central Hospital of Wuhan, Tongji Medical College, Huazhong University of Science and Technology, Wuhan, China; ^3^ Department of Burns, Tongren Hospital of Wuhan University, Wuhan, China

**Keywords:** intimal calcification, medial calcification, peripheral artery disease, atherosclerosis, arterial stiffness

## Abstract

Lower limb arterial calcification (LLAC) is associated with an increased risk of mortality and it predicts poor outcomes after endovascular interventions in patients with peripheral artery disease (PAD). Detailed histological analysis of human lower artery specimens pinpointed the presence of LLAC in two distinct layers: the intima and the media. Intimal calcification has been assumed to be an atherosclerotic pathology and it is associated with smoking and obesity. It becomes instrumental in lumen stenosis, thereby playing a crucial role in disease progression. On the contrary, medial calcification is a separate process, systematically regulated and linked with age advancement, diabetes, and chronic kidney disease. It prominently interacts with vasodilation and arterial stiffness. Given that both types of calcifications frequently co-exist in PAD patients, it is vital to understand their respective mechanisms within the context of PAD. Calcification can be easily identifiable entity on imaging scans. Considering the highly improved abilities of novel imaging technologies in differentiating intimal and medial calcification within the lower limb arteries, this review aimed to describe the distinct histological and imaging features of the two types of LLAC. Additionally, it aims to provide in-depth insight into the risk factors, the effects on hemodynamics, and the clinical implications of LLAC, either occurring in the intimal or medial layers.

## Introduction

Arterial calcification can occur in almost all vascular beds and linked with atherosclerosis. The prevalence of arterial calcification increases with age and is associated with traditional cardiovascular risk factors (1). Significant strides have been made in clinical studies on lower limb arterial calcification (LLAC) in the past decades and various evaluation methods have been proposed. It is regarded as a potential driver of peripheral artery disease (PAD) and is linked to increased cardiovascular events and death (2, 3). Additionally, it has been linked to poor outcomes following endovascular procedures and has been confirmed to be a better indicator of major amputation than the combined ankle-brachial index (ABI) and conventional cardiovascular risk factors taken together (4, 5). Currently, most research on LLAC is based on digital subtraction angiography (DSA) and Computed Tomography (CT), which offer a comprehensive view of calcifications and often used for auxiliary diagnosis and preoperative evaluation.

The coronary artery calcium score was originally used for evaluating atherosclerotic burden in the coronary arteries and is now commonly used as a quantitative approach to explore the clinical relevance of LLAC (6). Unlike coronary calcification, the histopathologic evidence reveals that LLAC predominantly involves medial calcification, which brings new perspectives on the significance of LLAC patterns in future clinical research (7). In this review, we document the distribution and histopathological features of LLAC, its available imaging methods for assessing LLAC, and its clinical relevance, which may contribute to a better understanding of PAD and calcification mechanism.

## Histological features and LLAC

The arterial wall can be divided into three layers: (from the inner layer to the outer layer): the intima layer, media layer, and adventitia. Vascular calcification is caused by the accumulation of disseminated minerals in the vascular system. According to the location of mineral deposits, it can be classified into intima and media calcification (1). As they are independent disease states driven by distinct pathophysiological mechanisms, it is essential to discuss them separately (8).

Intimal calcification is correlated with atherosclerosis and is known as the consequence of modified lipid accumulation, pro-inflammatory cytokines, and remodeling of extracellular matrix proteins in response to vascular injury (1). In the early stages of atherosclerosis, inflammation infiltrates lead to gradual thickening of the intima, accompanied by the formation of microcalcification (<5 um) (9, 10). Calcification initially appears in the injured vascular smooth muscle cells (VSMCs) either internally or externally, and is widely dispersed in the extracellular components, some of them are taken up by macrophage foam cells (11). After continuous fusion of adjacent granules, microcalcified deposits can form large structures (12). This process can be divided into three phases: initiation, propagation, and endstage (13, 14). In the initiation phase, macrophages precede calcification while releasing pro-osteogenic cytokines. In the propagation phase, the process is thought to be derived from apoptotic VSMCs, or matrix vesicles (“exosomes”) released by these cells near the internal elastic lamina. Both the calcifying matrix vesicles and apoptotic bodies may provide new nucleation sites, facilitating the precipitation of calcium salts at the micrometer scale (8, 15). The final phase is linked with little inflammation and marked tissue mineralization (15).

Media calcification differs from atherosclerosis that the pathophysiology is not fully understood. According to the existing research, medial calcification is considered to result from the osteogenic differentiation of VSMCs within the medial layer of the vascular wall (16). The pathophysiology is the phenotypic shift of VSMCs from a contractile to an osteochondrogenic phenotype. The phenotypic shift of VSMCs is typified by the absence of contractile markers and increased expression of bone-related genes: bone sialoprotein, bone morphogenetic protein, osteocalcin, transcription factors, Cbfa1/Runx2, Sox9 and Msh homeobox 2 (17, 18). Human peripheral arteries with medial calcification were shown to have elevated levels of osteo/chondrogenic markers (19). It is widely recognized that Runx2 serves as a major regulator mediating this phenotypic transition, not only by activating bone gene expression but also by inhibiting myocardi, which governs the expression of VSMCs markers (20).

Inflammation and specific genotypes also present a critical regulatory in the development of medial calcification. Inflammation indirectly influences the pathogenesis of medial calcification by impacting the signaling pathways involved in its pathogenesis (15, 21). Tumor necrosis factor-α (TNF-α) is the most important cytokine, involved in both bone and vascular physiology. Macrophages are the primary source of TNF-α synthesis after they are triggered by oxidized low-density lipoprotein (Ox-LDL), infection, or extracellular matrix decomposition products (22, 23). Its function in regulating VSMCs and osteoblast proliferation and differentiation is most likely modulated by the runt-related transcription factor Runx2 (24). Additionally, there is a monogenetic autosomal recessive disease closely resembling the manifestation of medial calcification. The lack of CD73, resulting in an increase in activity of tissue non-specific alkaline phosphatase, a critical protein in bone formation (25).

Intimal calcifications frequently present as thick, patchy and discontinuous clusters, predominantly affecting large arteries. In contrast, medial calcifications present as thin, circular, and continuous lesions and commonly seen in peripheral medium and small-sized arteries (26, 27). Histologically examinations conducted by Vos et al. on 270 postmortem samples from the tibial arteries revealed that the presence of medial calcification increases from the proximal popliteal artery to the more distal posterior tibial artery and most lesions are of the nonatheromatous type (28). In contrast, the presence of intimal calcification decreases from proximal to distal. This finding is consistent with pathological diagnosis. O'Neill et al. observed that amputation specimens of patients with PAD predominantly nonatheromatous fibrotic lesions and intimal thickening, with the frequency of atherosclerotic lesions were relatively low (7). These findings suggest that the pathophysiology of PAD is significantly different from the atherosclerotic process described in other arteries, while medial calcification may be the major type of vascular calcification in PAD.

## Imaging measurement on LLAC

**Figure 1:**
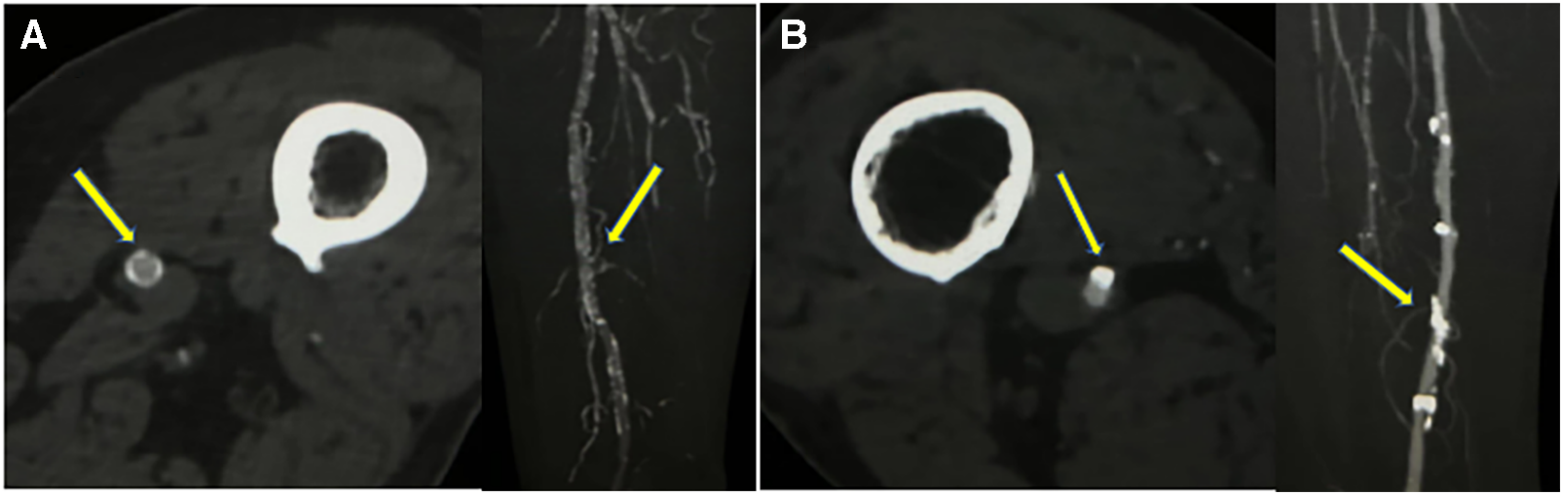
Two different superficial femoral artery calcification patterns on CTA (window width 1,300 Hounsfield Units; window level 300 Hounsfield Units) (**A**) axial CTA images showed calcifications <1.5 mm, >270° of artery circumference, maximal intensity projection showed continuous calcification (indicating dominant medial calcifications). (**B**) axial CTA images showed calcifications >1.5 mm, <90° of artery circumference, maximal intensity projection showed continuous irregular calcification (indicating dominant intimal calcifications).

Vascular calcification can be detected with various imaging techniques. Non-invasive imaging techniques, including x-ray, CT, magnetic resonance imaging (MRI), and ultrasound. In 1903, Mönckeberg first described medial arterial calcification (MAC) in radiography, where it appears linear and contiguous along the vessel edge, resembling rail tracks. In contrast, intimal arterial calcification (IAC) usually appears as irregular, spotty or discrete plaque. However, distinguishing between MAC and IAC using conventional CT images is challenging due to limited spatial resolution. To address this, a thin-slice CT imaging score was proposed to analyze the two types of carotid siphon calcifications based on three imaging features: annularity, thickness and continuity (26). One study showed that current radiography or CT is justifiable for detecting and characterizing both types of arterial calcification in large epidemiological studies when evaluated by experienced observers (Figure 1) (27). MRI is deemed unsuitable for calcification evaluations due to its sensitivity for calcification is lower, while recent advances in MRI can accurately identify plaque components, such as lipids, thrombus and fibrous tissue (29). Vascular imaging techniques based on ultrasound enable the detection of vascular calcification and the differentiation between MAC and atherosclerosis lesions. MAC lesions appear as distinct echogenic granules located in the abluminal layers of the arterial walls on ultrasound imaging (30). Intravascular imaging techniques, such as intravascular ultrasound (IVUS) or optical frequency domain imaging (OFDI) allow differentiation between the intimal and medial calcifications. On IVUS, MAC lesions present as hyperechoic areas located within the media. These lesions typically no no acoustic shadowing due to the presence of fibrotic tissue. Conversely, calcification in the intima often leads to acoustic shadowing (30). However, it is worth noting that both IVUS and OFDI have limitations in identifying MAC in overlapped intimal calcification or when the line between the intima and media is unclear (31). Moreover, due to the invasiveness and relatively high cost, it is used rarely in clinical practice.

In clinical, CT angiography (CTA) and DSA are considered common references to quantify and characterize LLAC (The assessment methods of LLAC can be usually divided into two categories, qualitative visual scores and quantitative methods like the Agatston score and calcium volume) (Table 1). Qualitative visual grading systems evaluate three main factors: morphology, location and extent of calcification through visual inspection. The most commonly used visual score is the peripheral artery calcification scoring system (PACSS) (16). The scoring was first proposed by Rocha-Singh KJ et al. and could be evaluated only by angiography. Qualitative visual scores are quick and simple but with some observers subjective. Additionally, some details in calcification might be missed and inconsistencies between different grades make it challenging to compare findings from different studies.

**Table 1:** Current methods for calcification measurement.

Method	Description
Qualitative measurement	
Characteristic (eg: Kockelkoren methods)	
Circularity	
Point 0	Absent
1	Dot(s)
2	<90 degrees
3	90–270 degrees
4	270–360 degrees
Thickness	
Point 0	Absent
1	Thick ≥1.5 mm
3	Thin <1.5 mm
Morphology	
Point 0	Indistinguishable
1	Irregular/Patchy
4	Continuous
PACSS	
Grade 0	Novisible calcium at the target lesion site
1	unilateral calcification <5 cm; (a) intimal calcification; (b) medical calcification; (c) mixed type
2	unilateral calcification ≥5 cm; (a) intimal calcification; (b) medical calcification; (c) mixed type
3	bilateral calcification <5 cm; (a) intimal calcification; (b) medical calcification; (c) mixed type
4	bilateral calcification ≥5 cm; (a) intimal calcification; (b) medical calcification; (c) mixed type
Quantitative measurement	
Agatston score	Calcification area of the arteries are identified in every slice with a density of more than 130 HU (Hounsfield units), multiplied by a cofactor that depends on the peak density of each plaque (130–199 HU = 1; 200–299 HU = 2; 300–399 HU = 3; ≥400 HU = 4), and total score is calculated by summing the respective values in the regions of interest.
Volume	Manual or semiautomatic measurement of calcified voxels depending on different threshold values

Characteristic (eg, Kockelkoren methods), <7, Dominant Intimal, ≥7, Dominant Non-Intimal.

Peripheral Arterial Calcification Scoring Systems (PACSS): Intimal and medial vessel wall calcification at the target lesion site as assessed by high intensity fluoroscopy and digital subtraction angiography (DSA) assessed in the AP projection.

In comparison, quantitative methods are reproducible and objective by using specific software. The Agatston score is a well-established and verified quantitative measure used to evaluate coronary arteries, which is defined as a weighted density assigned to its highest attenuation value multiplied by the area of calcification (32). However, since IAC and MAC can differ in prevalence and morphology, the misleading impact of the Agatston score cannot be ignored.

Above all, various methods are used for LLAC evaluation, including subjective visual assessments and objective quantitative evaluation. Because of the different standards utilized for grading LLAC, a reproducible and quantitative assessment method is urgently needed to facilitate comparisons between different studies.

## Clinical relevance of LLAC

### Risk factors

MAC and IAC represent distinct pathophysiologies that correlate with traditional cardiovascular risk factors in different ways (1, 33). As both forms of calcification in the majority of PAD patients, it is hard to assess the proportional impact of specific risk factors to their development.

MAC is most commonly associated with age, diabetes and chronic kidney disease (CKD) (34–39). It is recognized as an age-associated pathology (39). The aged VSMCs have been correlated with the upregulation of molecules that drive osteogenic differentiation (40, 41). It was suggested that aged VSMCs might play a key role in driving osteo/chondrogenic change and calcification in PAD (40, 41). Diabetes and CKD are considered inflammatory conditions. The TNF-α, inflammatory cytokine, and inflammation-induced oxidative stress have been shown to contribute to arterial calcification linked with diabetes by promoting pro-calcific Msx2-Wnt signaling cascades (42, 43). Additionally, metabolic disorders in diabetes and CKD lead to vascular mineralization. High serum phosphate levels not only facilitate the precipitation of hydroxyapatite crystals but also modulate critical signaling pathways central to MAC pathophysiology: the phenotypic shift of VSMCs to osteoblast-like cells, VSMCs apoptosis, extracellular matrix remodeling, and the suppression of monocyte/macrophage differentiation into osteoclast-like cells (44, 45). Multiple overlapping mechanisms by which diabetes could result in arterial calcification were proposed: inflammation, hyperglycemia, advanced glycation end products, circulation of osteoprogenitor cells, and reduced levels of matrix gamma carboxy glutamic acid protein (46–49).

Studies on IAC, report positive associations with smoking and dyslipidemia (35, 50). Nicotine found in tobacco promotes vascular calcification, and its mechanisms involve the hyperactivation of inflammatory responses, oxidative stress, autophagy, increased cell apoptosis, extracellular matrix degradation, and disruption of endothelial function (51–53). LDL causes the formation of atherosclerosis through various mechanisms, including the upregulation of inflammatory cytokines and adhesion molecules on vascular endothelial cells, increased transport of oxPLs, inhibition of nitric oxide synthesis, resulting in vascular remodeling, and promotion of smooth muscle cell proliferation and foam cell formation (54).

These studies highlight the various risk factors that contribute to the complex pathophysiology of LLAC and offer valuable practical guidelines for the therapeutic and preventive management of LLAC.

### Hemodynamic effects of LLAC

Intimal calcification is associated with atherosclerotic luminal stenosis, a critical factor in the genesis of territorial hypoperfusion. In the intracranial arteries and coronary arteries, the clinical and pathologic studies demonstrated a significant correlation between calcium burden and occlusive disease (55–57). In the lower extremities, two studies used CTA to investigate the relationship between calcium burden and the severity of lumen stenosis. Zettervall et al. found that LLAC was related to the severity of ischemia in patients with PAD and had shown a moderate correlation with the severity of occlusive disease. However, there was a weak correlation when included only those patients with critical limb-threatening ischemia (CLTI) (58). This phenomenon could be caused by vascular calcification of PAD is mostly located in the media and might even be more obvious in CLTI (7). Yan et al. further found that the LLAC of the aortoiliac artery was relatively weak (36). One possible mechanism the iliac artery had a relatively large diameter, making it less affected by the calcium volume of the vessel wall. Nevertheless, the retrospective design of these studies and their quantitative measurements of LLAC negatively impact the reliability of identifying a connection between LLAC and vascular stenosis. Large prospective studies using more precise measurement tools are still needed to confirm the correlation between LLAC and vascular stenosis.

Historically, MAC is thought to be limited to the media and not associated with luminal stenosis (28). However, the accumulation of calcification within the media layer can lead to vascular deformation and the extension of calcified plaques into the lumen. Additionally, medial calcification can also lead to subendothelial hyperplasia, causing nonatheromatous intimal thickening and resulting in luminal stenosis (30, 59). A study in patients with CKD showed that there was a connection between the severity of MAC in abdominal arteries and carotid intima–media thickness (60). Although it happens in different vascular beds, this may indicate that MAC loading of the vascular wall can result in adverse remodeling. In both diabetic and nondiabetic limbs, Ferrier observed a strong correlation between advanced MAC and significant obstruction of the metatarsal artery (61). In small vessels, which are disproportionately influenced by MAC, this can lead to catastrophic consequences on the lumen cross-section area, including the potential pruning of pedal end arteries with far-reaching adverse effects on tissue perfusion (62).

MAC caused a significant change in the vessel wall structure and function. The accumulation of calcium deposits in the media between the elastic fiber induces arterial stiffening, resulting in degradation of compliance and vasodilation restriction (63, 64). Nonetheless, while the adverse impact of MAC in the aorta on stiffening, systolic blood pressure, and coronary perfusion has been extensively researched, the pathological effect of MAC in other vascular beds is poorly understood (65, 66). The peripheral arterial system stiffens in PAD with MAC. This will probably influence localized blood flow, and it has also been suggested that it may exacerbate the consequences of aortic stiffness and affect brain and heart health (67). Pulse wave velocity is a diagnostic tool for peripheral arterial disease and a measurement of arterial stiffness, but it does not offer direct insights into the anatomical characteristics or biological processes of atherosclerotic plaques (21, 68). Further research is needed to define these mechanisms.

As mentioned before, IAC and MAC coexist in patients with PAD (69). By restricting positive vascular remodeling and promoting negative one, MAC can have an impact on atherosclerosis (70). Additionally, it can cause subendothelial hyperplasia, which can aid in the development of atherosclerotic plaque (30). Consequently, when MAC and atherosclerosis coexist in the same artery segment, the signs of PAD are more severe and develop more quickly.

### Plaque vulnerability

The relationship between calcification and acute coronary artery embolism has been confirmed (71, 72). In patients with CLTI, the histopathology in the lower limb arterial is characterized by luminal occlusion of the lower leg vascular and is accompanied by inconspicuous atherosclerosis (73). One study indicates that the presence of calcium deposit is independently and inversely associated with acute thrombosis in patients with PAD (74). Due to the most widely used imaging technologies (CT and DSA) cannot differentiate between medial and intimal calcium deposits, making it challenging to assess their specific contribution to arterial thrombosis development.

A histopathological investigation of 60 patients with CLTI who had amputation reported a 5.6% prevalence of arterial thrombotic occlusion. Interestingly, 70% of the specimens were predominantly MAC without significant atherosclerotic plaque (7). Another study examining arterial specimens from 95 patients who had amputation reported that 67.5% of the samples had occlusive thrombosis without significant atherosclerotic plaque (73). The same study revealed that MAC-related thrombosis was more prevalent in the inferior knee arteries, whereas atherosclerotic plaque rupture-related thrombosis was more common above the knee. The possible mechanism is that medial calcification leads to blood stasis due to loss of pulsatile blood flow, which increases the risk of arterial thrombosis (21). This suggests that the mechanism of arterial occlusion in PAD may be more related to thromboembolism than thrombosis at the site of plaque rupture as commonly reported in coronary artery disease (73).

### Clinical significance of LLAC

LLAC is commonly observed in patients with PAD, and it has been linked to the severity of PAD symptoms (58). A growing interest among researchers in the clinical significance of LLAC is due to findings indicating that severe calcification independently correlates with major amputation and mortality. Methods for evaluating LLAC are not yet unified, and different studies use different measurement methods. Guzman et al. first used agaston score to assess calcification in the tibial artery (4). In patients with PAD, they found that the highest tibial arterial calcification scores were linked to worsening levels of limb ischemia and it identifies high-risk populations for amputation. Building on this scoring method, Chowdhury et al. evaluated LLAC and found that higher scores were an independent predictor for cardiovascular events and all-cause mortality (3).

Treatment PAD includes endovascular revascularization and open surgical operations. The available data show that LLAC limits the success of peripheral arterial revascularization. In patients with femoropopliteal artery occlusions, Itoga et al. demonstrated that 100% calcification on preoperative CT was a predictor of technical failure of endovascular revascularization (75). Due to the small vessel caliber, tibial artery occlusion is difficult to analyze. Kang et al. used the semiquantitative analysis of CTA to categorize calcification severity in tibial arteries and found that extensive calcification is also a predictor of technical failure in the tibial artery region (5). Consequently, the success rate of revascularization is lower in patients with severe calcification and improvement is often short-term, leading to a late lumen loss. In 2014, a study showed that the presence of annular calcium distribution seems to be the strongest predictor of optimal drug absorption when drug-eluting balloons are used (76). Tepe et al. retrospective study used PACSS scoring to show grade 4 was significantly correlated with 6-month primary patency after drug-coated balloons angioplasty for femoropopliteal lesion (77). Additionally, circumferential calcification was strongly correlated with a less favorable late lumen loss. The reasons for increased late lumen loss in severely calcified lesions are as follows. Firstly, the biological efficacy of drug-eluting balloon treatment relies on adequate drug transfer and accumulation in the target artery. However, the presence of annular calcification in the arterial wall may be an impermeable barrier for the drug, thus diminishing efficacy of the therapy and bare metal stent use was associated with better patency outcomes (78, 79). Second, annular calcification reduces vascular compliance and may lead to subacute vascular recoil, triggering increased late lumen loss. Moreover, above studies received different treatment methods, including stents, atherectomy, or drug-eluting balloons, the observations would not be applied to all types. Future studies need to compare the outcomes of calcification between common treatment modalities in larger samples to draw firmer conclusions.

LLAC can not only serve as a predictor of success rate of revascularization procedures and greater restenosis but also impact the prognosis after revascularization therapies, including function outcome and postintervention mortality. Megale et al. showed that the LLAC scores are linked to an increased risk of mortality, regardless of the revascularization method used (80). To better assess and predict limb-related outcomes, various clinical scoring systems have been developed. LLAC evaluation can further stratify the existing clinical scores. Huynh et al. established a scoring system focusing on calcification morphology, circumference, and length in the common iliac artery, found that incorporation of this score into the Vascular Quality Initiative mortality prediction model improved risk stratification for patients who were initially classified as lower-risk group risk for mortality (81). The Society for Vascular Surgery WIfI (wound, ischemia, foot infection) system stratifies the risk of amputation, independent of the vascular anatomy. The global limb anatomic staging system (GLASS) enables assessment of the anatomic complexity and target artery pathway for surgical intervention (82, 83). Nevertheless, the relative contribution of arterial occlusive disease below the ankle is not known. The MAC has been most frequently described in the foot artery plaque lesions (84). Recently, a study by Liu et al. used foot x-rays to develop a simple scoring system to assess pedal artery calcification in patients with CLTI undergoing infrainguinal revascularization (85). Higher pedal calcification scores were correlated with a high risk of major amputation. These studies not only establish a clear relationship between MAC and adverse limb outcomes, but also demonstrate the potential of the MAC score to complement the current standard of noninvasive tests, WIfI staging, and GLASS in constructing a more comprehensive view of each patient's disease process, anatomy, and evolving limb threat in addition to better direct the therapy of patients with CLTI. Further large-scale studies are needed to verify and compare these results.

## Conclusions and future perspectives

LLAC is an important facet of PAD and a marker of overall disease burden. Although the role of different LLAC patterns in PAD is currently much better known than in previous decades, some significant problems connected to different LLAC patterns remain to be solved. These are the prevention and therapeutic approaches and visualization methods.

Although IAC and MAC share common risk factors, but there are some differences in the remaining risk factors (37, 38). Therefore, it is expected that the efficacy of measures used in the treatment and prevention of different LLAC patterns differs. IAC is associated with atherosclerosis, the primary therapeutic goal focused on the treatment of local disease and prevention of related systemic cardiovascular complications (86). LDL levels are highly correlated with cardiovascular outcomes (87). Only one study has shown a connection between LDL and PAD outcomes as in coronary artery disease (CAD) (88). In patients with aging, diabetes, and CKD, in whom media calcification is significantly more prevalent (37). Although the pathogenesis is better understood, there is still no effective treatment and preventive strategy for MAC (21). The current way relies on the existing risk factors, which means that there is no specific treatment. Further studies to elucidate the pathophysiology of MAC will probably reveal new treatment and preventive targets.

Quantification and visualization are also crucial aspects in the assessment of LLAC. Currently, most of the research models on lower extremity calcification are based on simulations from coronary and carotid arteries, but the relationship between lower extremity lesion characteristics and therapeutic outcomes compared to coronary and carotid arteries is more intricate. Moreover, the ability of noninvasive tools to differentiate between IAC and MAC and quantify MAC is limited. In the future, the utility of noninvasive ways could be improved by integrating a well-designed software that enables automatic quantification of calcium deposition in specific vascular regions and the estimation of the relative contribution of IAC/MAC to the overall calcium burden.
